# The biomass–density relationship in seagrasses and its use as an ecological indicator

**DOI:** 10.1186/s12898-018-0200-1

**Published:** 2018-10-19

**Authors:** Vasco M. N. C. S. Vieira, Inês E. Lopes, Joel C. Creed

**Affiliations:** 1 MARETEC, Instituto Superior Técnico, Universidade Técnica de Lisboa, Av. Rovisco Pais, 1049-001 Lisbon, Portugal; 2 Departamento de Ecologia, Instituto de Biologia Roberto Alcântara Gomes, Universidade do Estado do Rio de Janeiro, Rua São Francisco Xavier 524, Rio de Janeiro, RJ 20559-900 Brazil

**Keywords:** Ecosystem, Seagrass, Coastal, Index, Meadow, Nutrient, Pollution

## Abstract

**Background:**

Biomass–density relations have been at the centre of a search for an index which describes the health of seagrass meadows. However, this search has been complicated by the intricacy of seagrass demographics and their complex biomass–density relations, a consequence mainly of their modular growth and clonality. Concomitantly, biomass–density upper boundaries have been determined for terrestrial plants and algae, reflecting their asymptotic maximum efficiencies of space occupation. Each stand’s distance to its respective biomass–density upper boundary reflects its effective efficiency in packing biomass, which has proved a reliable ecological indicator in order to discriminate between taxonomic groups, functional groups and clonal vs. non-clonal growth.

**Results:**

We gathered data from 32 studies on 10 seagrass species distributed worldwide and demonstrated that seagrasses are limited by their own boundary line, placed below the boundaries previously determined for algae and terrestrial plants. Then, we applied a new metric—d_grass_: each stand’s perpendicular distance to the seagrass boundary—and used this parameter to review fundamental aspects such as clonal growth patterns, depth distribution, seasonality, interspecific competition, and the effects of light, temperature and nutrients.

**Conclusions:**

Seagrasses occupy space less efficiently than algae and terrestrial plants. Using only their biomass and density data we established a new and efficient tool to describe space occupation by seagrasses. This was used with success to evaluate their meadows as an ecological indicator for the health of coastal ecosystems.

**Electronic supplementary material:**

The online version of this article (10.1186/s12898-018-0200-1) contains supplementary material, which is available to authorized users.

## Background

**Fig. 1:**
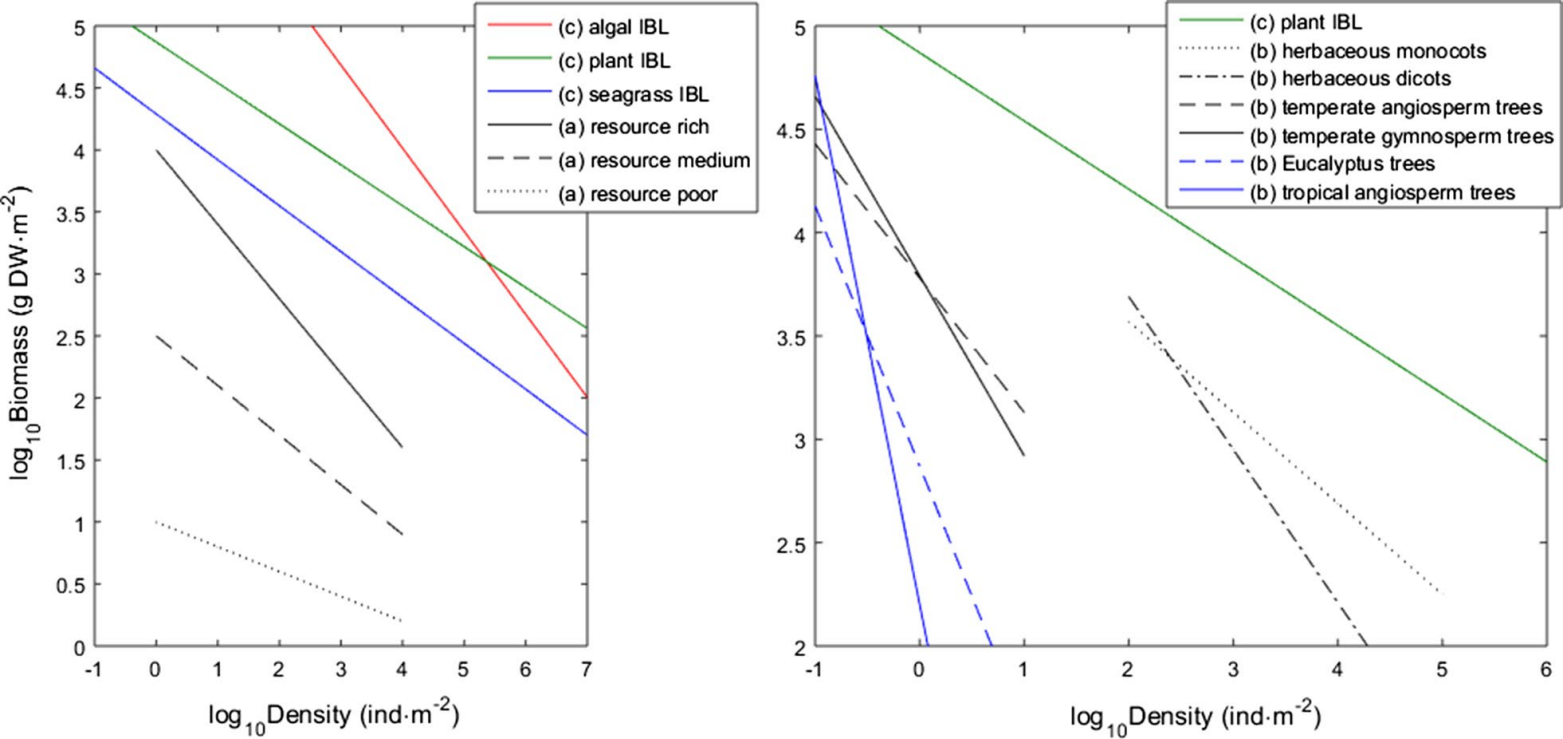
Biomass–density relationships. The trajectories in (a) are a schematic of the generalized observed pattern with the stands’ specific relationships dependent from resource availability. These were not drawn from observed data nor represent any specific taxon. The trajectories in (b) are taxon specific Boundary Lines drawn from data of Weller [9]. The trajectories in (c) are the interspecific boundary lines (IBL) of plants [20], algae [12] and seagrass (estimated in this study)

When individuals in an even-aged monospecific plant stand undergo active growth increasing their biomass, competitive stress may induce mortality. The consequent elimination of the weaker plants releases resources (space, light and nutrients) facilitating the further growth of survivors. This dynamic, commonly known as self-thinning, also reflects the efficiency of space occupation as more efficient stands (or species) exhibit higher biomasses under similar stand densities (numbers of individuals per unit area). The first studies of self-thinning, dating from the 1950s onwards [1–4], established a relationship between density (D) and mean plant mass (w) given by *w *= *kD*^−3/2^ or equivalently log_10_*w *= log_10_*k *− 1.5 log_10_*D*. Here, *w* refers to above-ground biomass and *k* is an allometric constant. In addition to many reported self-thinning slopes in plants ranging from small herbs to trees being close to -3/2, the law has also been applied to some mixed species stands [5] and across species regressions of different sized plants [6, 7]. Later the relationship evolved into an equivalent derived from stand biomass per unit area (*B*) and density: *B *= *kD*^−1/2^ or equivalently log_10_*B *= log_10_*k *− 0.5log_10_*D* [8]. This new relation solved two problems: (i) auto-correlation, as the former *w*-*D* relation required the number of individuals to estimate the quantities on both sides of the equation, and (ii) mean biomass increasing without actual growth but just because smaller individuals died [8, 9]. This was the first improvement aiming at establishing a set of best-practices for the assessment of biomass–density relations and involving aspects such as the data quality and choice of regression methods [9, 10]. More recently, the numerical aspects were further improved with the development and application of model 2 quantile regression and careful data screening [11, 12]. These adjustments brought about the realization that the slope of the biomass–density regression should be reset at values closer to *−* 0.33 [10, 13]. Despite these advances, substantial debate followed as to what the self-thinning “law” is as well as controversy as to whether a law really existed at all [10, 14, 15]. Henceforth, biomass–density relationships were categorized into three different aspects (Fig. 1):

- The (intraspecific) dynamic self-thinning line is the straight line that is approached, then followed by the time trajectory of a crowded monospecific stand as it grows [9, 10, 13–15]. Stands may have different dynamic thinning lines depending on the environmental conditions (= carrying capacity). Changes in the slope and intercept of this dynamic thinning line for stands of the same species usually relate to the allometry of a plant species [16] as well as to resource or temperature limitation [17–19]. In the later cases, flatter slopes associated with lower intercepts reflected smaller carrying capacities of the environment.
- The species (or higher ranked taxon) boundary line—the upper boundary of possible biomass–density combinations for a given taxon from the plant kingdom [15]. This line is fit to the most extreme density-biomass combinations of hundreds of stands of the same taxon. Theoretically, the y-intercept of a taxon provides information about its maximum capacity to pack biomass above-ground.
- The interspecific biomass–density relationship (IBDR [20]) and its static upper boundary characterizing the maximum biomass–density limit for all the species which make up the plant kingdom. Weller [13] analysed plant data setting the boundary at log_10_B = 3.91 − 0.33 log _10_D. However, this boundary was estimated from Ordinary Least Squares (OLS) and thus, although applied to the most extreme stands, it still determined a central tendency. Consequently, Scrosati [20] re-analysed the data from Weller [13] setting the plant boundary at log_10_B = 4.87 − 0.33 log_10_D and dubbing it ultimate biomass–density Line (UBDL) [21]. More recently, Creed et al. [12] termed this boundary the interspecific boundary line (IBL).

Since the 1980s phycologists have tested the biomass–density relations in seaweeds, generally obtaining results similar to those obtained from land plants [22–27]. However, a recent analysis of data revealed that algae occupy space more efficiently [12]. With a higher intercept and steeper slope (log_10_B = 6.69 − 0.67 log_10_D), the algal IBL was placed significantly higher than the plant IBL (Fig. 1), thus representing the ultimate boundary known for any life-type on the planet. The higher efficiency of algae was considered to be a consequence of the aquatic environment which facilitates the algal morphology and ecophysiology relative to that of plants. Compared to land plants, algae are neither limited by water availability nor bare costs of transporting water for transpiration; they also acquire nutrients and photosynthesize through the whole of their surfaces, and do not require (non-photosynthetic) deep roots or rigid tissues for support as buoyancy keeps them upright.

Seagrasses grow by the iteration of modules which may remain physiologically integrated or separate to form clones. Previously it has been shown in plants and algae that the intraspecific dynamic biomass–density relationship (self-thinning) often does not apply to clonal species. These may have clonal integration because modules (ramets) belong to the same genetic individual (genet) and are physically interconnected, which may allow the sharing of acquired resources between ramets and offset competition [28]. Consequently, an increase of stand biomass may arise from the increase in plant (ramet) sizes, increase in plant (ramet) density, or both. Hutchings [28] identified several non-thinning biomass–density dynamics typical of clonal plants. On the other hand, Westoby [29] and de Kroon and Kalliola [30] identified clonal plants where the specificities of their life-histories may (or not) lead them to self-thin, depending on additional factors. The occurrence of self-thinning is also variable in clonal algae and seems to depend on their specific life-history, morphological characteristics and habitat [20, 31–34]. Although not necessarily self-thinning, terrestrial clonal plants and clonal algae were nevertheless demonstrated to be limited by an IBL [12, 28]. Thus, it was still possible to use their stand’s distance to their IBL as an estimator of their efficiency of space occupation. Having proved applicable to all sorts of algae, both clonal and non-clonal, this new method provided exciting new evidence about algal growth and ecology [12]. In this work we tested whether the same applies to seagrasses. Taxonomically, seagrasses are flowering plants (i.e., angiosperms), sharing with their terrestrial counterparts the features that make angiosperms the most complex taxa among autotrophic life-forms: as well as having flowers they also possess rigid tissues, extensive roots and a primary circulatory system carrying water and nutrients. On the other hand, they spend a significant part (if not all) of their life underwater, where they can also benefit from the water environment, as do macroalgae. Furthermore, in contrast to terrestrial angiosperms, seagrasses acquire nutrients both through their leaves and roots, a capacity that can even become essential for their metabolic rates [35]. Given this seagrass duality, the first question arising is whether seagrasses pack above-ground biomass similarly to algae and/or terrestrial plants?

The dynamic self-thinning line is thought to depend intrinsically on the plant taxon being considered, besides being dependent extrinsically on the environmental carrying-capacity (proposed by Weller [9, 13, 16]). Biologically more complex plants, appearing later in Life’s evolutionary history and generally comprehending larger individuals (namely trees), showed significantly steeper slopes along with lower intercepts (Fig. 1). Backed by elementary biostatistics, ecological theory proposed the intercept as a standard to estimate the biomass carrying capacity under equivalent densities. Hence, Weller’s results suggested that simpler plants occupy space (i.e., pack above-ground biomass) more efficiently. Weller’s [9] finding was refuted by Lonsdale [10] on alleged methodological grounds. Recently, new evidence emerged corroborating Weller’s findings. Creed et al. [12] also found that the algal efficiency of space occupation varies with algal traits, namely with taxonomic group, functional group and clonality. And similarly to Weller’s analysis simpler taxa occupy space more efficiently. Concurrently, the ability of plant communities to pack biomass into the volume effectively exploited has been demonstrated to depend on biodiversity and on the efficiency of resource exploitation [36]. Creed et al. [12] obtained their results using a more straightforward and robust methodology than the one used by Weller [9] with each stand being measured by its perpendicular distance to the algal IBL (d_algal_). Following the results obtained by Weller [9], Proulx et al. [36] and Creed et al. [12], in this study we use the perpendicular distance to the seagrass IBL (d_grass_) to test whether different species show different efficiencies of space occupation in seagrasses too.

As seagrasses occur at the interface of land and sea they suffer diverse stressors, one of which is eutrophication [37]. Increasing nutrient loadings are potentially toxic and also promote blooms of opportunistic macrophytes [38]. Both effects have a negative impact on biomass or density in seagrass stands that may extend to the whole ecosystem [37–42]. Seagrasses more impacted by algal competitors tended to be smaller and/or less abundant (see Fig. 2 in Thomsen et al. [40]). As seagrasses are foundation species the dynamics of their stands has been used as a proxy for ecosystem health [39, 43–47]. The recent methodology by Creed et al. [12], which congregates the biomass and density data into a single metric irrespective of their specific correlation, estimates the stands’ efficiency of space occupation. We test here whether this metric applied to seagrasses is a meaningful ecological indicator. Do healthier seagrass stands better optimize their above-ground space occupation by falling closer to a biomass–density boundary line?

## Methods

### Biomass–density data retrieval

We gathered data of shoot density and above-ground biomass from 32 studies on 10 seagrass species distributed worldwide (Table 1). Most of the *Halodule wrightii* data was provided by one author (J.C.C.). The data from Plus et al. [48] was provided by Dr Martin Plus. The remaining data was retrieved from the respective publications using appropriate software. The compilation of data was carried out during years 2017 and 2018, and used the Google search engine as well as the search engines in the webpages of all cited publications. The search keywords included ‘biomass’, ‘density’, ‘seagrass’ and the species scientific denominations. We also searched the publication listings of the most cited authors in the subject and the reference lists of the cited works.

**Table 1:** Meta-data used for the seagrass biomass–density relation

Species	Sources	No. obs.	Location	Latitude
*Cymodocea nodosa*	Agostini et al. [72]	12	Urbinu lagoon, Corsica	42.02
*Cymodocea nodosa*	Duarte and Sand-Jensen [71]	47	Ebro Delta, Spain	40.72
*Cymodocea nodosa*	Sghaier et al. [75]	36	Monastir Bay, Tunisia	35.37
*Cymodocea nodosa*	Peduzzi and Vukovic [74]	17	Golf of Trieste, Italy	45.7
*Cymodocea nodosa*	Cunha e Duarte [73]	5	Ria Formosa, Portugal	37.10
*Halodule wrightii*	Hall et al. [60]	12	Florida Bay, USA	25.14
*Halodule wrightii*	Creed (this study)	992	Multiple sites, American continent	–
*Posidonia oceanica*	Terrados and Pons [76]	5	Magaluf, Mallorca Island, Spain	39.30
*Posidonia oceanica*	Terrados and Pons [76]	5	Ses Salines, Mallorca Island, Spain	39.15
*Posidonia sinuosa*	Keulen [77]	14	Shoalwater bay, Queensland, Australia	− 22.42
*Posidonia sinuosa*	Collier et al. [78]	18	Cockburn & Warnbro sounds, Western Australia	− 32.17
*Posidonia sinuosa*	Fraser and Kendrick [79]	45	Cockburn & Warnbro sounds, Western Australia	− 32.17
*Syringodium filiforme*	Hall et al. [60]	2	Florida Bay, USA	25.14
*Thalassia hemprichii*	Larsson [70]	3	Inhaca & Portuguese Islands, Mozambique	− 25.9
*Thalassia testudinum*	Hall et al. [60]	197	Florida Bay, USA	25.14
*Thalassia testudinum*	Tamasko and Hall [64]	56	Charlotte Harbour, Florida, USA	26.9
*Thalassia testudinum*	Galegos et al. [65]	30	Cancún, Mexico	21
*Thalassia testudinum*	Enríquez and Pantoya-Reyes [67]	9	Puerto Morales, Cancún, Mexico	20.87
*Thalassia testudinum*	Paynter et al. [68]	3	Punta Cahuita, Costa Rica	9.7
*Thalassia testudinum*	Kaldy and Dunton [66]	20	Laguna Madre, Texas, USA	26.13
*Thalassia testudinum*	Medina-Gómez et al. [69]	6	Bahia de la Ascencion, Mexico	19.7
*Zostera japonica*	Lee et al. [49]	18	Dadae Bay, Geoje Island, Korea	34.43
*Zostera japonica*	Ruesink et el. [55]	20	Stackpole, Willapa Bay, Washington, USA	46.59
*Zostera japonica*	Ruesink et el. [55]	20	Oysterville, Willapa Bay, Washington, USA	46.54
*Zostera japonica*	Ruesink et el. [55]	20	Nahcotta, Willapa Bay, Washington, USA	46.49
*Zostera marina*	Lee et al. [49]	18	Dadae Bay, Geoje Island, Korea	34.43
*Zostera marina*	Olesen and Sand-Jensen [62]	32	North America, Europe & Japan	30 to 56
*Zostera marina*	Kim et al. [57]	46	Seomjin Estuary, South Korea	34.9
*Zostera marina*	Krause-Jensen et al. [56]	766	Oresund strait	55.6
*Zostera marina*	Möller et al. [61]	9	Prangli, Baltic Sea, Finland	59.63
*Zostera marina*	Möller et al. [61]	7	Sõru, Baltic Sea, Finland	58.69
*Zostera marina*	Möller et al. [61]	2	Saarnaki, Baltic Sea, Finland	58.80
*Zostera marina*	Möller et al. [61]	3	Ahelaid, Baltic Sea, Finland	58.74
*Zostera marina*	Ruesink et el. [55]	4	Stackpole, Willapa Bay, Washington, USA	46.59
*Zostera marina*	Ruesink et el. [55]	5	Oysterville, Willapa Bay, Washington, USA	46.54
*Zostera marina*	Ruesink et el. [55]	5	Nahcotta, Willapa Bay, Washington, USA	46.49
*Zostera marina*	Jones et al. [80]	34	British Isles and Nothern Ireland	50.6 to 54.6
*Zostera nolti*	Cabaço et al. [43]	276	Ria Formosa, Portugal	37.10
*Zostera nolti*	Cabaço et al. [45]	16	Ria Formosa, Portugal	37.10
*Zostera noltii*	Garcia-Marín et al. [46]	13	Ria Formosa, Portugal	37.1
*Zostera noltii*	Garcia-Marín et al. [46]	13	Huelva, Spain	37.2
*Zostera noltii*	Garcia-Marín et al. [46]	13	Cadiz, Spain	36.5
*Zostera noltii*	Plus et al. [48]	54	Thau Lagoon	43.4
*Zostera noltii*	Plus et al. [63]	22	Thau Lagoon	43.4

### Biomass–density data analysis

From the 2954 biomass-shoot density observations available we selected the 500 closer to the top right corner of the seagrass biomass–density plot. With these 500 observations we estimated a seagrass-specific IBL following the procedure described by Creed et al. [12] and choosing the 99.9% quantile for the linefit. Once this boundary was known, we estimated the stands’ perpendicular distances to this line (the d_grass_ presented in “The d_grass_ metric” section below) in order to test:

i. How seagrasses worldwide have different efficiencies of space occupation. However, the data comprised species with widely different numbers of observations, which would inevitably bias comparisons based on properties of the samples’ distributions. In an attempt to overcome this problem and simultaneously compare among seagrasses at their maximum efficiency, for this task we selected from each taxon the five observations with the smallest d_grass_;
ii. How *Z. marina* and *Z. japonica* in Dadae Bay show different efficiencies of space occupation while inhabiting the same shoreline;
iii. How the efficiency of space occupation varies seasonally and spatially;
iv. How the efficiency of space occupation varies with light, temperature and nutrient concentrations.

### The d_grass_ metric

**Fig. 2:**
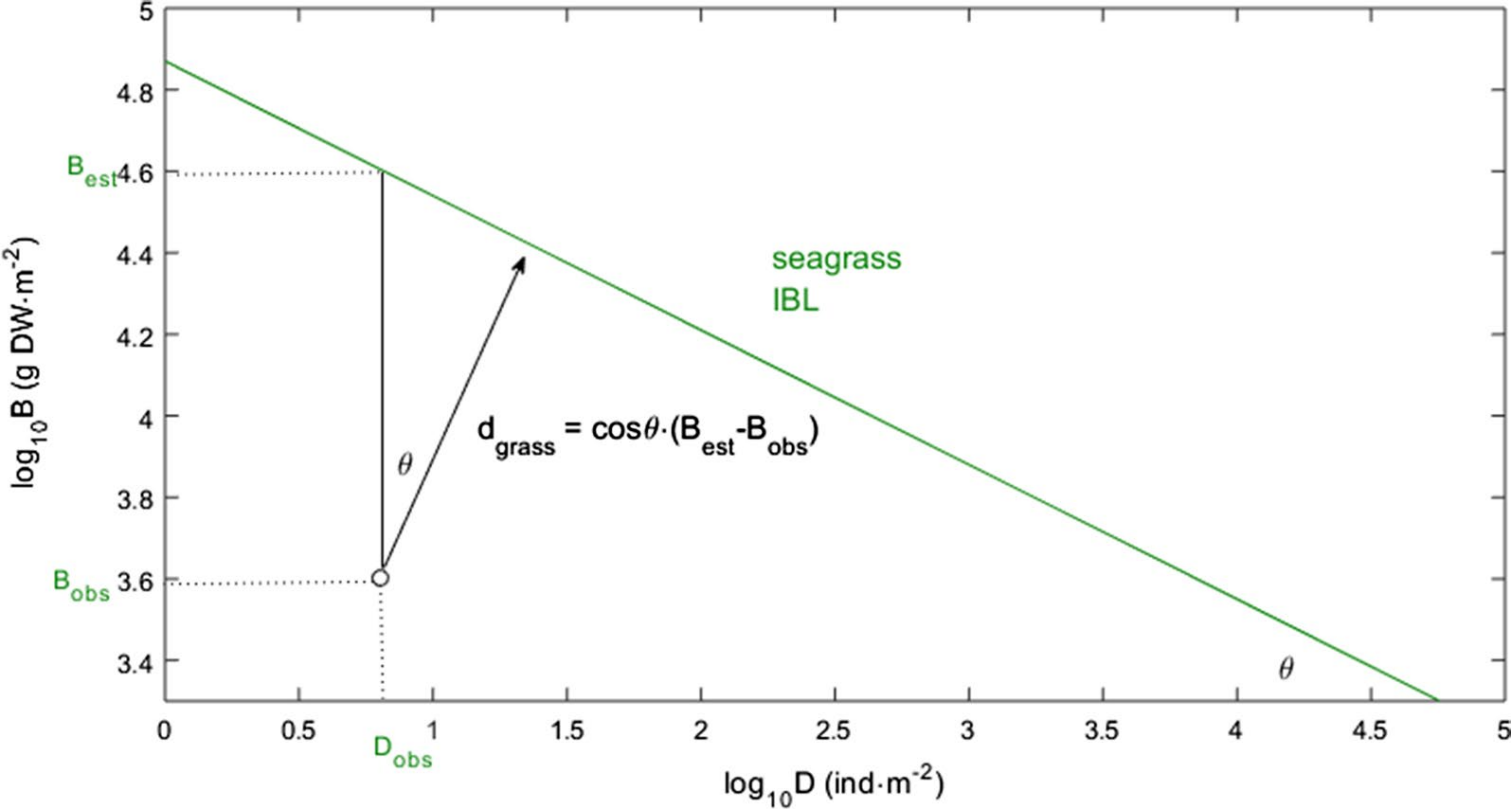
Estimation of the perpendicular distances (d_grass_). These are estimated from the observed (obs) and estimated (est) biomass (B) and density (D), and the seagrass IBL

The estimation of each stand’s perpendicular distance to the seagrass IBL (d_grass_) requires the linear coefficients of this boundary line. With the general IBL equation corresponding to log_10_B = β_0_ + β_1_ log_10_D, the coefficients for the seagrass IBL were β_0_ = 4.569 and β_1_ = − 0.438 (as presented in “Results” and “Discussion” sections). The angle θ between the d_grass_ vector (oblique in the log_10_B-to-log_10_D orthogonal plane) and the log_10_B vertical axis is the same angle between the seagrass IBL and the log_10_D horizontal axis (Fig. 2). Hence, θ = arctg(|β_1_|), which in the seagrass case corresponds to θ = 0.413. The perpendicular distance is the cosine of θ, in the case of seagrass cosθ = 0.916, multiplied by the B vertical distance: d_grass_ = (log_10_Ḃ − log_10_B)∙cosθ. The vertical distances require the observed log_10_B and the estimated log_10_Ḃ = β_0_ + β_1_∙log_10_D.

### Additional data

The biomass–density data was correlated with additional biotic and abiotic data to test which factors may determine seagrasses demography. These additional data were taken from the studies from which the biomass–density data was taken (Table 1). The full dataset is provided as additional files.

Lee et al. [49] compared the demography of the *Zostera marina* and *Zostera japonica* native to the South Korean coast. The *Zostera marina* occupied the subtidal whereas the *Zostera japonica* occupied the intertidal. Sampling occurred from July 2001 to July 2002, and retrieved a set of biotic variables related to the populational, morphometric and ecophysiological properties of the seagrasses, and another set of abiotic variables characterizing the water properties, sediment properties and light regime.

Plus et al. [48] studied factors determining the primary production of *Zostera noltii* in the Thao lagoon from July 1996 to August 1998. The biotic variables retrieved included biomass and density. The abiotic variables included water temperature, salinity, photosynthetic active radiation (PAR) at the surface and on the bottom, light extinction coefficient, and the concentrations of NH_4_^+^, NO_2_^−^, NO_3_^−^ and PO_4_^3−^.

Stands of *Zostera noltii* were followed in the Ria Formosa lagoon system in southern Portugal at four locations along a pollution gradient [45]. Site 1 was the closest to the wastewater treatment plant (more polluted—270 m away). At the opposite extreme, site 4 was the farthest from the wastewater treatment plant (little polluted—1500 m away and located in the main channel). All sites were simultaneously assessed during July 2001 (summer), November 2001 (autumn), February 2002 (winter) and May 2002 (spring). To this data we added data from the *Z*. *noltii* studied by Plus et al. [48] in the Thau Lagoon.

Aiming for an indicator of water quality and ecosystem health, Garcia-Marín et al. [46] developed the ZoNI index from compiled data for intertidal *Z. noltii* stands from the southern Iberian Peninsula. Five stands were sampled in the summer of 2010 in Ria Formosa, south Portugal, three of them assumed under normal conditions (R1, R2 and R3) and two of them impacted by extreme pollution from urban effluents (I1 and I2). Three stands were sampled both in Huelva (H1, H2 and H3) and in Cádiz (C1, C2 and C3), south Spain, during the summers of 2009–2011. Biotic variables included biomass, density, and other populational, morphometric and biochemical characteristics. Abiotic variables included ammonium, nitrate and phosphate concentrations, and the first principal component (PC1) extracted from a principal components analysis (PCA) synthesized the nutrients general dynamics.

## Results

**Fig. 3:**
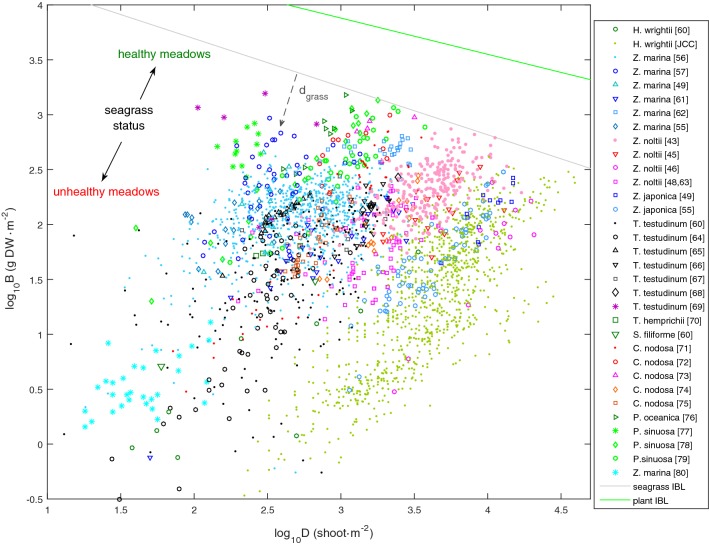
Seagrass biomass-shoot density relations worldwide. Biomass (B) and shoot density (D) of seagrasses, their interspecific boundary line (IBL) given by log_10_B = 4.569 − 0.438∙log_10_D, and stands’ distances to the IBL (d_grass_). Status of seagrass meadows was labelled as ‘healthy seagrass meadows’ inhabiting favourable environments, and ‘unhealthy seagrass meadows’ inhabiting less favourable environments

**Fig. 4:**
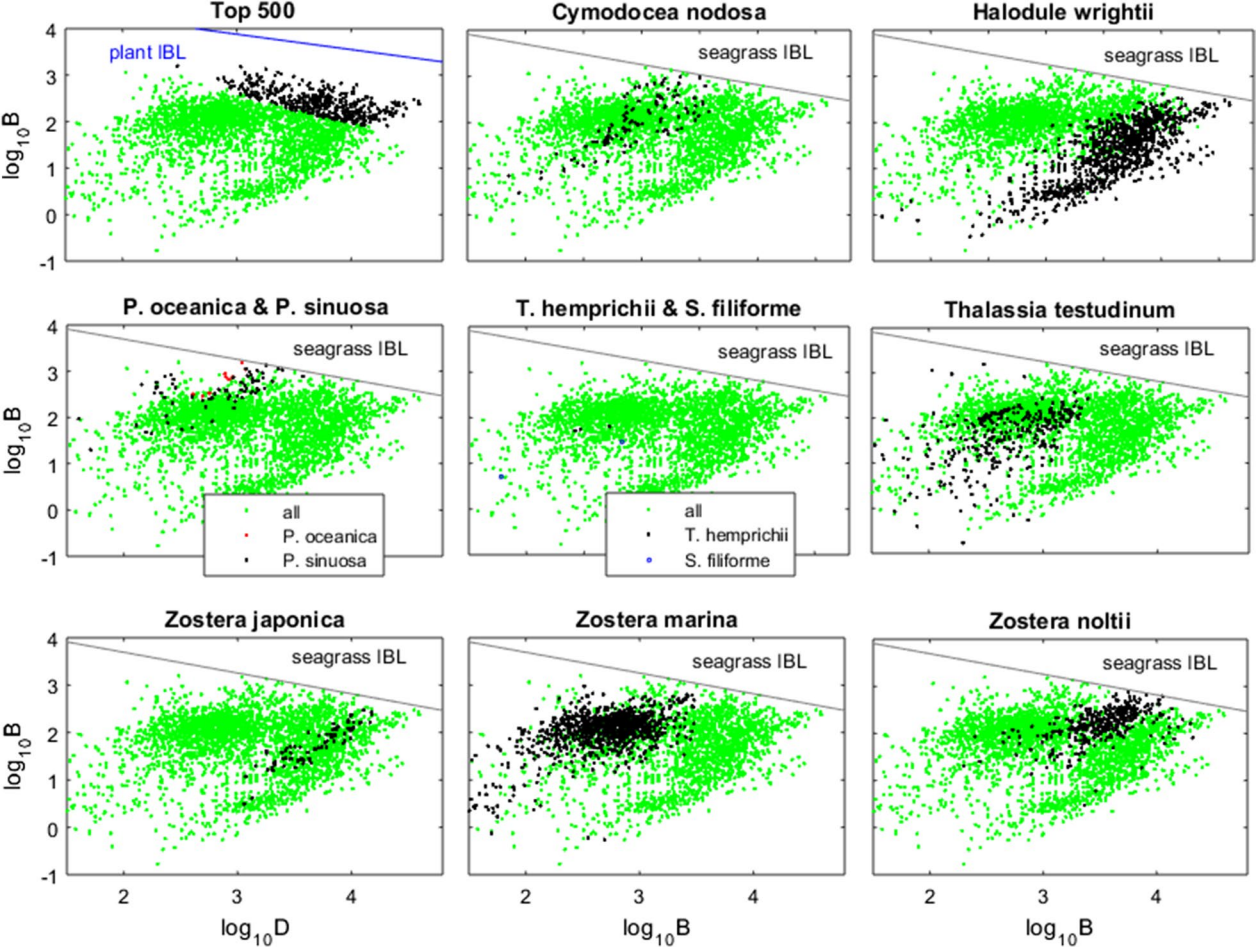
Biomass-shoot density relations specific of each taxon. Green markers. All seagrass observations; black markers—selected seagrass observations

**Fig. 5:**
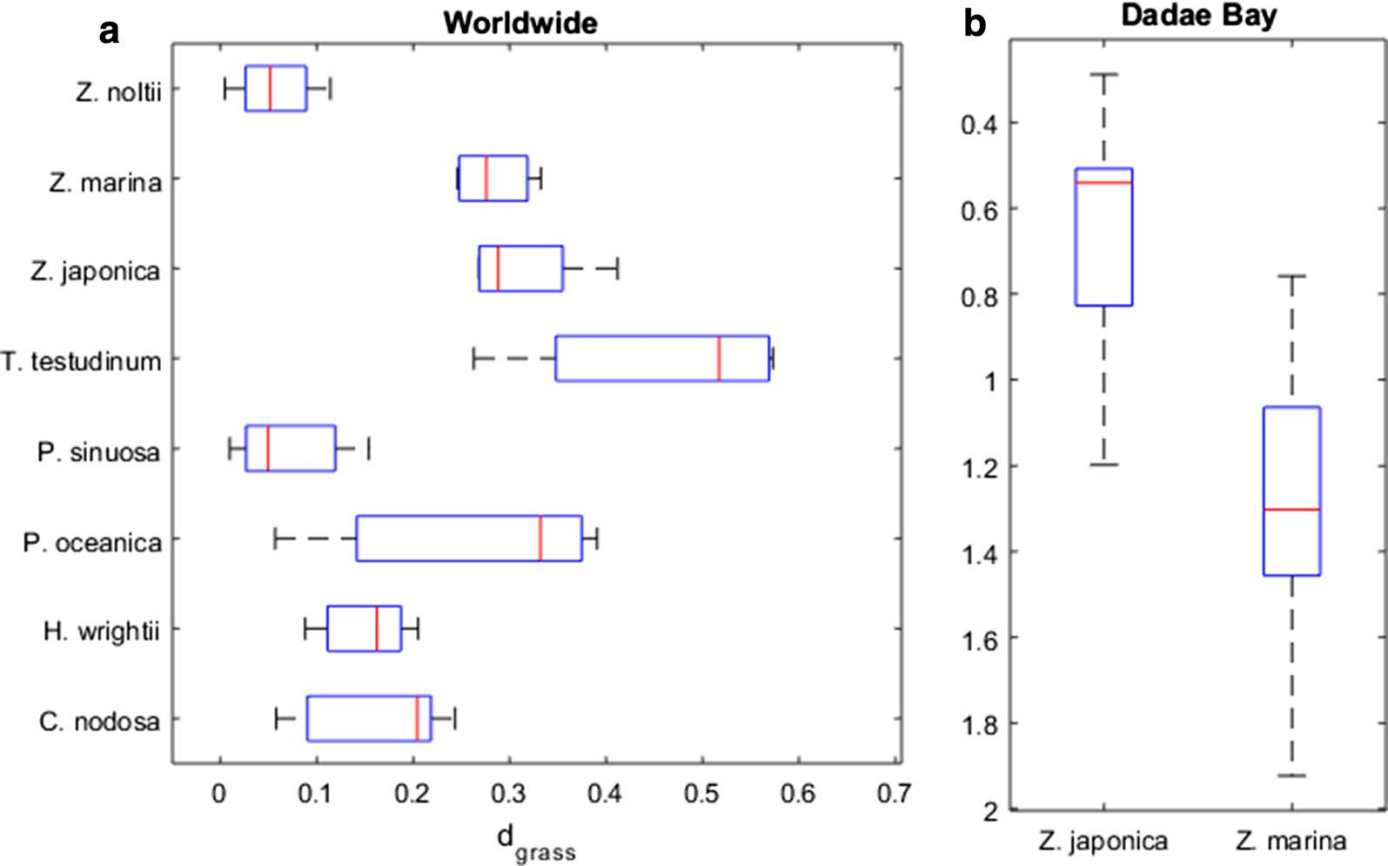
Seagrass discrimination by efficiency of space occupation. Each stands’ distances to the seagrass IBL (d_grass_) is used as a measure of this efficiency. This measure was compared **a** among taxa worldwide and **b** for the case study of Dadae Bay [49]. Box and whiskers represent the quartiles of the sample distribution

Similarly to algae and terrestrial plants, seagrasses also had their specific biomass–density boundary (Fig. 3). With a slope of –0.438 and an intercept of 4.569, the seagrass boundary (IBL) placed far below that of the plant and algae boundaries. This lower placement was mainly due to the lower B-axis intercept. Below their own IBL, seagrasses often showed a positive correlation between their biomass and shoot density, while differentiating among themselves by occupying different bands of the biomass-shoot density spectra (Figs. 3 and 4). The five best efficiencies observed for each species showed that different seagrasses have different maximum efficiencies of space occupation (Fig. 5a). Permutation tests (a class of non-parametric ANOVA where the null hypothesis is simulated by randomly redistributing the data) with 10,000 replications showed that these maximum efficiencies were significantly different (p = 0.0001, d.f._error_ = 40, d.f._groups_ = 7).

Stands of *Z. marina* and *Z. japonica* at Dadae Bay showed similar biomasses, though *Z. japonica* achieved this under much higher densities of smaller shoots (shown in Fig. 3), resulting in a much better space occupation efficiency (Fig. 5b). The Dadae Bay situation seems to be case-specific, suggesting the occurrence of something particular, as the worldwide trend is for both species to show similar efficiencies of space occupation (Fig. 5a).

**Fig. 6:**
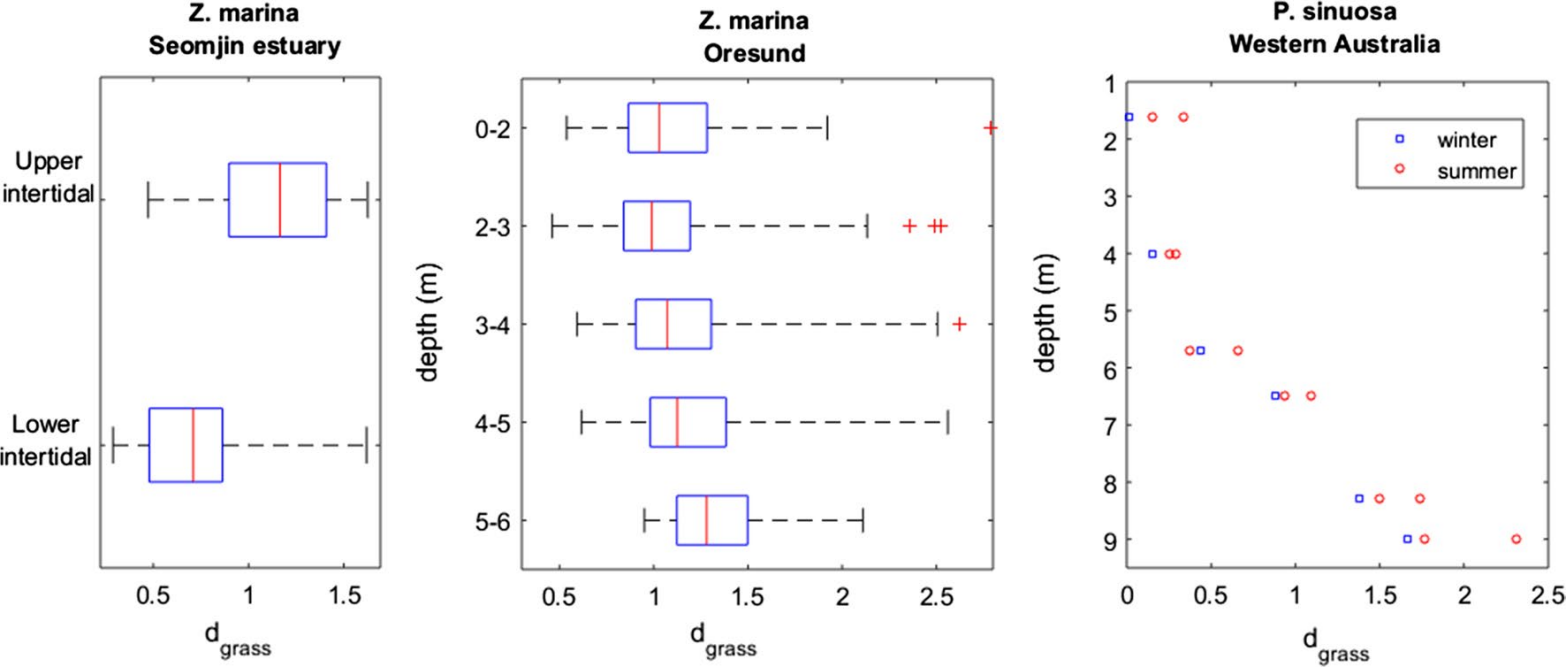
Effect of depth on the efficiency of space occupation of seagrasses. Each stands’ distances to the seagrass IBL (d_grass_) is used as a measure of this efficiency. *Zostera marina* was sampled in the Seomjin estuary—South Korea [57] and in the Oresond strait, Baltic Sea [56]. *Posidonia sinuosa* was sampled in Cockburn Sound and Warnbro Sound, Western Australia [78]. Box and whiskers represent the quartiles of the sample distribution, and asterisks represent outliers

The efficiency of space occupation by *Zostera marina* in the Baltic and by *Posidonia sinuosa* in Western Australia decreased with depth (Fig. 6). For the *P. sinuosa* in Western Australia a seasonal trend was also observed where the efficiency of space occupation increased in the winter and decreased in the summer (Fig. 6). This seasonal pattern opposes the pattern observed for the other species and is presented as follows.

**Fig. 7:**
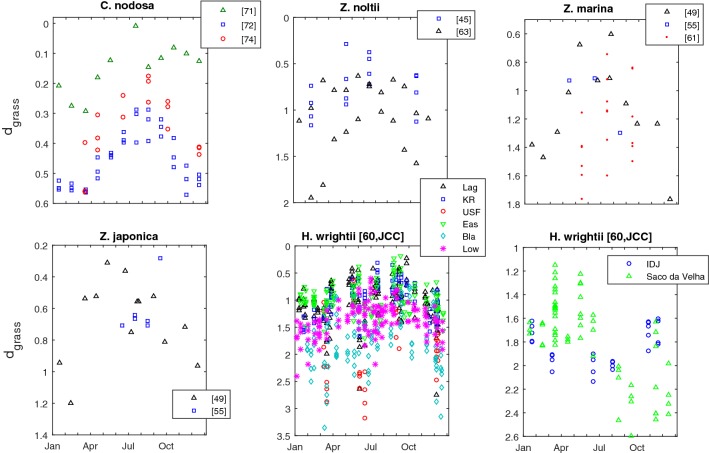
Seasonality of the seagrass efficiency of space occupation. The seagrass efficiency of space occupation is evaluated from the distance to the seagrass IBL (d_grass_)

The d_grass_ metric confirmed the general seagrass seasonality but also highlighted regional differences (Fig. 7). The seagrasses from higher latitudes within the northern hemisphere, namely *Cymodocea nodosa* and the *Zostera* spp., peaked their efficiency of space occupation during the spring–summer and reached the low point during the winter. Located closer to the equator, the *Halodule wrightii* stands in the northern hemisphere peaked during June–September (summer). One of the stands located in the southern hemisphere peaked during November-January (summer) whereas the other slightly delayed its peak to the summer-autumn. Concomitantly with the seasonal dynamics, the d_grass_ also discriminated between stands at different locations experiencing different environments. This was particularly evident in the *C. nodosa* and the *H. wrightii* populations.

**Fig. 8:**
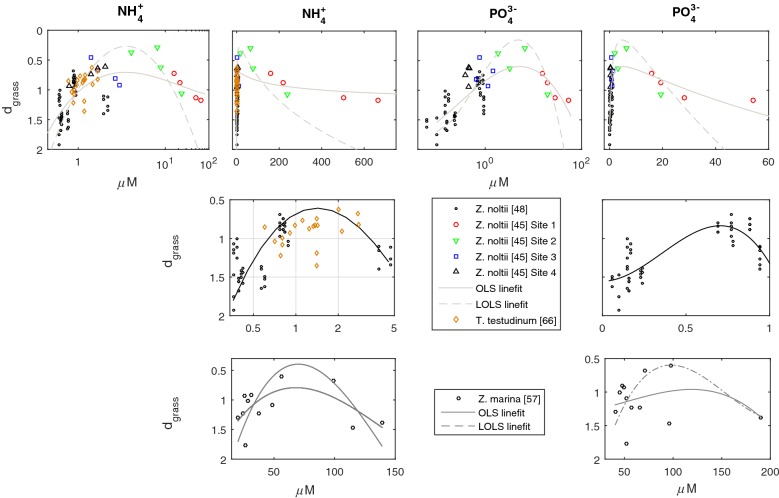
Effects of amonium and phosphate on the efficiency of space occupation (d_grass_) by *Zostera noltii* and *Thalassia testudinum* stands. Stands in Ria Formosa are in a gradient of closeness to a wastewater treatment plant, and measured seasonally. Site 1 was the closest and more polluted and site 4 the furthest away and least polluted [45]. Stands in the Thau Lagoon sampled by Plus et al. [48]. *T. testudinum* stands sampled by Kaldy and Dunton [66].  Model fits by Ordinary Least Squares (OLS) and Linear-in-the-parameters Oblique Least Squares (LOLS) [81]. The LOLS was fit by the new debuged software provided as Additional file 3

The effects of ammonium and phosphate on the efficiency of space occupation peaked at intermediate concentrations, mimicking the dynamics generally reported for the effects of these nutrients on the growth rates of seagrasses (Fig. 8). The optimal nutrient concentrations for *Z*. *noltii* in Ria Formosa were much higher than in the Thau Lagoon. The *Z*. *noltii*’s most efficient space occupation at Ria Formosa were found at the sampling sites located at intermediate distances from the wastewater treatment plant. The concentrations of other nutrients showed no effects over the *Z. noltii* stands in the Thau Lagoon and in Ria Formosa (Fig. 8).

In the Thau lagoon, temperature and PAR at the bottom (i.e., at stand level) were the fundamental factors governing *Z. noltii*’s efficiency of space occupation. The d_grass_ was better fit to the congregation of a quadratic function for temperature and another quadratic function for PAR (Eq. 1), although the resulting forth order term was disregarded from the numerical estimation of coefficients.

1 $$d_{grass} = \left( {a_{0} + a_{1} T + a_{2} T^{2} } \right)\left( {b_{0} + b_{1} PAR + b_{2} PAR^{2} } \right)$$

**Fig. 9:**
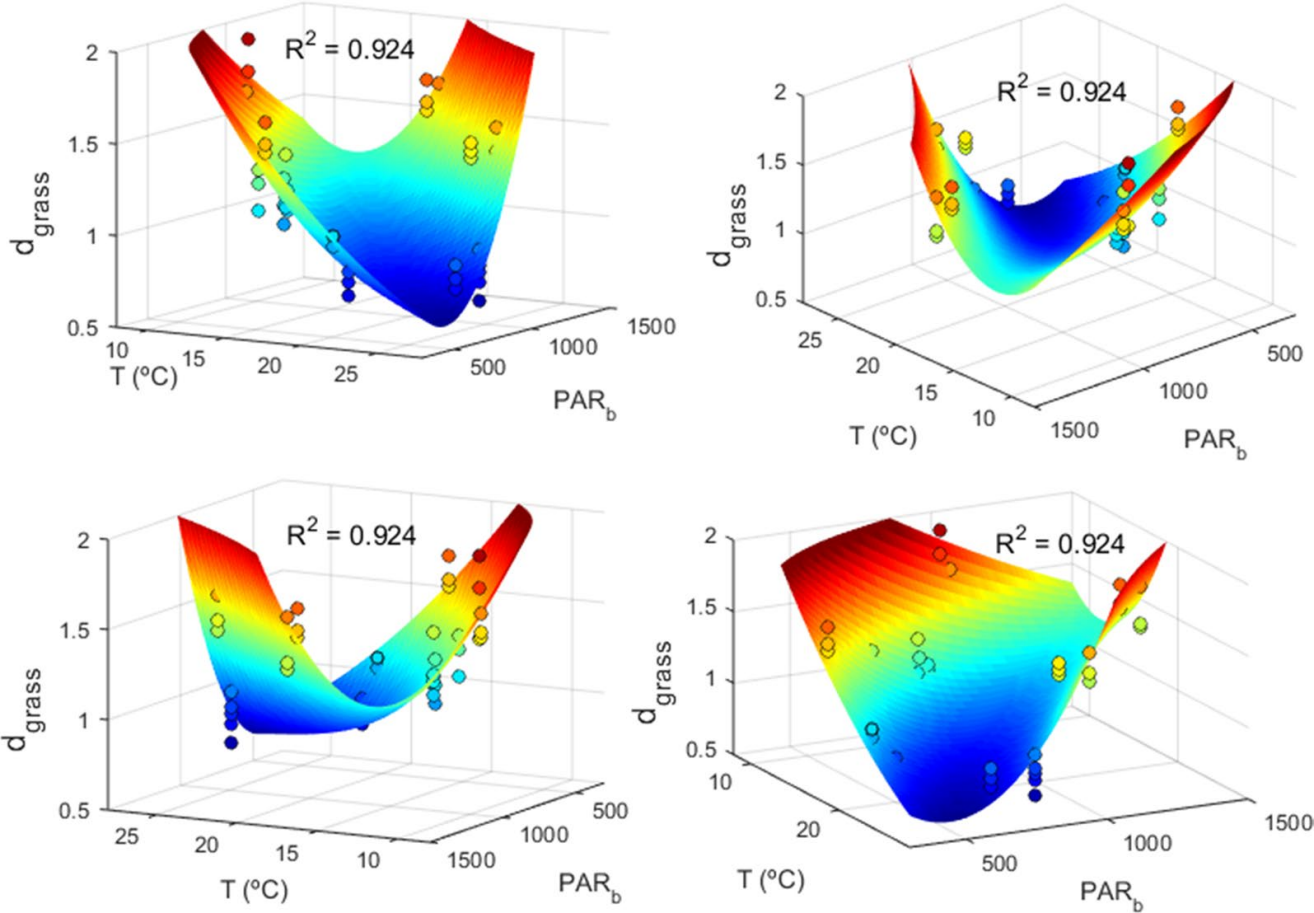
Abiotic drivers of the efficiency of space occupation (d_grass_) by *Zostera noltii* in the Thao lagoon. The d_grass_ of stands sampled by Plus et al. [48] is dependent from temperature (T) and photosynthetic active radiation at the bottom (PAR_b_). The d_grass_ fit was performed by Ordinary Least Squares (OLS). All four panels are different perspectives of the same 3D plot showing the surface fit by Eq. (1)

The efficiency of space occupation (i.e., d_grass_) mimicked the photosynthetic and growth responses to irradiance as modelled by the classical photosynthesis-irradiance (P–I) curves. Hence, given a fixed temperature, there was an optimal PAR above and below which the space occupation deteriorated i.e., d_grass_ increased (Fig. 9). Concomitantly, given a fixed PAR, there was an optimal temperature above and below which the space occupation deteriorated (Fig. 9). The optimal temperature and optimal PAR were negatively correlated—i.e., as temperature increased its correspondent optimal PAR decreased—suggesting that temperature had a positive effect on the photosynthetic efficiency.

**Fig. 10:**
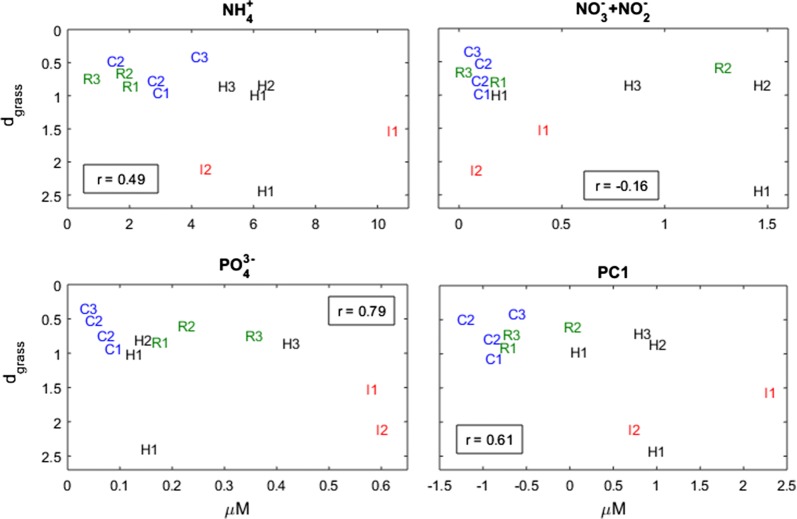
The d_grass_ of *Zostera noltii* stands in southern Iberia. Stands from Ria Formosa, Portugal in natural (R) or highly impacted (I) locations, from Huelva (H) and from Cádiz (C). Correlation coefficients (r) estimated disregarding H1 sampled during 2011

The ZoNI index correlated remarkably well with the d_grass_ (r = 0.92, see Additional file 1: Fig. S1). Correlating the d_grass_ with the abiotic data from Garcia-Marin et al. [46] (Fig. 10), it was found that Huelva’s stand 1 (H1) during 2011 showed an extremely weak space occupation as a consequence of some unaccounted factor, and hence was disregarded. The analysis on the remaining stands revealed that the fundamental constrain to *Z. noltii* space occupation was phosphate pollution above 0.5 μM. The polluted stands (I1 and I2) correlated phosphate concentrations above this threshold with an inefficient occupation of space (i.e., very large d_grass_). All other stands were below this phosphate concentration threshold and showed much smaller and similar d_grass_. Ammonium and nitrate had no influence on the inefficient space occupation by the polluted sites. In fact, these sites were even within the group of stands with lower nitrate concentrations. The correlation between d_grass_ and the nutrients PC1 was largely contaminated by the noise introduced in the nutrients PC1 by the presence of the ammonium and nitrate variables.

## Discussion

The lower placement of the seagrass IBL—due to its lower B-axis intercept—shows that seagrasses are poor occupiers of space, both compared to terrestrial plants, with whom they share taxonomic similarities, and when compared to algae, with whom they share the marine environment. The classical biomass–density models [1, 13] determine that one of the causes for a lower intercept is less volume exploited per unit plant stand surface (the other being less biomass per used volume). There are three possible causes for this event:

i. Wasted available volume due to short shoot heights, and thus also smaller height-to-width ratio. This constitutes vertical waste;
ii. Wasted available volume due to internode lengths larger than shoot widths. This constitutes horizontal waste that is fixed along the vertical axis;
iii. Wasted available volume due to shoot shape, as was similarly argued for the crown shape of trees [12, 50]. This constitutes horizontal waste that is variable along the vertical axis.

The scarcity of data with the required metrics did not allow generalization as to which aspects of seagrass allometry mentioned above affect their efficiency of space occupation. Nevertheless, the hypothesis (i) seems the most likely as seagrass canopies are relatively short when compared to their terrestrial counterparts. In permanently immersed stands, the shoots usually do not extend to fully occupy the available height (to sea-surface). Hence, it is unlikely that it is the sea-surface that usually acts as a barrier for vertical growth of seagrasses. A possible explanation for the height limitation comes from the fact that seagrasses generally lack secondary metabolites with anti-fouling properties frequently found in algae and hence need to avoid build-up of epiphytes which reduce light and nutrient uptake. They do so by using a basal meristem and changing leaves regularly, which puts a limit on both leaf length and biomass accumulation. The hypothesis (ii) about shoots narrower than internode length is unlikely, particularly if the stands’ have intermingled genets and/or branched rhizomes. Relative to hypothesis (iii) land crown shapes are less efficient at occupying space due to the structural adaptations of terrestrial plants for gravity. In water, less constrained by gravity, algae can use more efficient shapes, ultimately leading to an algae IBL placed above that of plants [12]. Immersed seagrasses are similarly less constrained by gravity, but shoot shape is constrained by leaf number per shoot and leaf width, which are relatively non-plastic seagrass variables, so space occupation depends on leaves elongating and presenting themselves horizontally. It should also be noted that seagrasses may not benefit as much from the support offered by the aqueous medium as seaweeds do because they still present some limiting structural tissue of their terrestrial ancestors (see Creed et al. [12] on this topic). Compared to their terrestrial counterparts, in these marine plants there may be a trade-off between the benefit of reduced investment in structural tissue and disadvantage of increased light attenuation. Despite their inefficient space occupation, seagrasses can dominate the intertidal and shallow subtidal in estuarine and lagoon systems, leading to the conclusion that the efficiency of space occupation is not the characteristic that concedes their ecological advantage over their algal competitors. As most algae need hard substrate to attach to, the fact that seagrasses can colonize soft sandy bottoms is the most likely explanation for their ecological advantage.

Different seagrasses showed different maximum efficiencies of space occupation. However, these estimates were very sensitive to the placement of the seagrass IBL, which in turn was estimated based only on ten species for which data are available. Furthermore, in the case of *Z. japonica* it is possible that its maximum efficiency, estimated lower than many other species, results from insufficient sampling. On the contrary, *H. wrightii* was sampled at 25 stations scattered along both tropical bands in the northern and southern hemisphere totalling 1005 observations, whereas *Z. marina* was sampled at 23 stations scattered worldwide and totalling 908 observations. Hence, although we should be cautious analysing these results, it seems safe to say that *Z. noltii*, *P. sinuosa*, *H. wrightii*, and *C. nodosa* are able to occupy space more efficiently than *T. testudinum*, *Z. japonica* and *Z. marina*.

Different seagrasses occupy different bands of the biomass-shoot density spectra, suggesting conditional differentiation of co-occurring seagrass species. As an example, *Z. marina* co-occurs with *Z. noltii* in the Atlantic and with *Z. japonica* in the Pacific Ocean; where it occupies a biomass–density band conspicuously different from those of its competitors. Within their respective bands, seagrass species tend to show a positive biomass–density correlation contrasting with the negative correlation typical of non-clonal plants and algae. This positive correlation typical of seagrasses is a consequence of their clonality [28] and suggestive of the growth-form plasticity already demonstrated in clonal terrestrial plants [51–53] and clonal algae [54]: the high shoot densities match the phalanx growth-form suited to dominate favourable environments [51]. On the other hand, when the environment is not favourable, the low shoot densities match the guerrilla growth-form allowing for faster dispersion in the search for better locations [51]. By mimicking the positive biomass–density correlation of seagrasses, the perpendicular distance to the seagrass IBL (i.e., the d_grass_), besides being an index for the efficiency of space occupation, is also an index for the trade-off in clonal-growth-form plasticity: small d_grass_ corresponding to high efficiencies and phalanx growth, and large d_grass_ corresponding to low efficiencies and guerrilla growth. This dynamic may seem paradoxical at first sight: when the environment is sub-optimal, adopting the guerrilla growth form gets the stand further away from the efficient space occupation, and thus more susceptible to competitors. On the other hand, when the environment is favourable, the phalanx growth form allows for the stand to reach the maximum efficiency more quickly, but at the cost of decreasing the stand biomass potential. It intuitively seems advantageous when competing with other seagrasses but not when competing with algae that have much higher space occupation efficiencies (see Creed et al. [12] for the algal IBL). Regarding this unorthodox phalanx strategy, we highlight three aspects:

i. Seagrasses avoid competition from algae by colonizing a substrate to which most algae cannot attach and grow;
ii. Thomsen et al. [38] found that small seagrasses (consequently, with lower biomasses and higher densities) are more vulnerable to their algae competitors;
iii. The advantage of reaching the boundary more quickly may also be related to complementary aspects. For instance, reaching the maximum biomass quicker may release resources for other aspects of seagrass development such as sexual reproduction, rhizome storage or growth at the edges of the stand.

The *Z. marina* and *Z. japonica* stands at Dadae Bay [49] are a good example of how seagrasses make use of phenotypic plasticity to adapt to the abiotic and biotic conditions, and how this affects their efficiency of space occupation. Both species had equally long leaves but the *Z. marina* leaves were wider. Furthermore, *Z. marina* showed a higher leaf production rate, leading to a higher number of leaves per shoot [49]. The consequent larger biomass per shoot of *Z. marina* relative to *Z. japonica* is a global attribute. However, at Dadae Bay it was fully compensated by the higher shoot density of *Z. japonica*, a consequence of its shorter internode length [49] and/or intermingled genets, and which led *Z. japonica* to occupy space more efficiently. The *Z. japonica* in Dadae Bay occupied the intertidal, which is its preferred environment [49, 55]. Hence, this stand adopted a phalanx growth-form whose high density and closeness to the IBL disables the colonization by its seagrass competitors. As for *Z. marina*, the application of the d_grass_ to the data taken from the studies by Krause-Jensen et al. [56] and Kim et al. [57], demonstrated that, worldwide, *Z. marina* is more efficient in the lower intertidal. However, in Dadae Bay the lower intertidal was already occupied by *Z. japonica*. Hence, *Z. marina* was relegated to the shallow subtidal where it adopted a guerrilla growth-form whose focus is on spreading to its preferred neighbouring lower intertidal. Contrasting the phalanx grow form of *Z. japonica* with the guerrilla grow form of *Z. marina*, the *Z. marina* internode length was on geometric average 2.5 times longer than that of *Z. japonica* while its reproductive effort was approximately 3 times higher (Additional file 2: data and Lee et al. [37]).

The efficiency of space occupation revealed itself to be an outstanding tool for understanding aspects of seagrass ecology. Based on this efficiency, we developed the d_grass_ metric, whose application to seagrasses worldwide enabled us to generalize regarding seasonality, their depth profiles, and their response to nutrients, light and temperature. We have already done the same for algae, developing the d_algal_ metric and applying it with success [12]. In the case of seagrasses, the d_grass_ seasonality matched the generalized summer peaks of seagrass growth and Photosynthesis–Irradiance (P–I) curve parameters [37]. The effects of ammonium and phosphate concentrations on d_grass_ mimicked the dynamics generally reported for the effects of these nutrients over the growth rates: very low concentrations were detrimental as autotrophs need nutrients to survive and grow. However, at high concentrations these molecules became toxic, shifting their role from nutrients to pollutants [39–41, 43–46]. The optimal nutrient concentrations for *Z*. *noltii* in Ria Formosa higher than in the Thau Lagoon suggested that some additional factor was influencing the optimal points. The *Z*. *noltii*’s most efficient space occupations in Ria Formosa located at intermediate distances from the wastewater treatment plant revealed that the wastewater often had a beneficial effect over those patches by raising the ammonium and phosphate concentrations to optimal values. The *Z*. *noltii*’s d_grass_ response to irradiance mimicked a P–I curve with photo-inhibition above the optimal irradiance, as has been reported for seagrasses [37] and for *Z. noltii* in particular [58]. Concomitantly, the *Z*. *noltii*’s d_grass_ response to temperature mimicked the previously reported parabola-type relation between seagrass growth and temperature [37].

We take the remarkable correlation of the ZoNI index with the d_grass_ as evidence that both are good indicators of the health of seagrass meadows. Monitoring seagrass health has been proposed for coastal water quality assessment [43–47]. The development of an ecological indicator relying exclusively on the biomass–density relation of seagrasses has been previously attempted, unsuccessfully [47, 59]. At that time, the existence of a seagrass IBL and the fact that the efficiency of space occupation is a fundamental aspect of seagrass ecology were unknown. Hence, back then it was impossible for the respective authors to succeed in their objective. Nevertheless, our new findings demonstrate that this is a useful concept and tool, which we put back on track reformulated around the efficiency of space occupation. We present three strong arguments for its future application:

i. *Its simplicity* The d_grass_ only requires two variables—the stand’s above-ground biomass and shoot density—which are also the easiest to retrieve and describe well habitat availability and complexity;
ii. *Its generality* It is applicable to any seagrass (estimating d_grass_), any terrestrial plant (estimating d_plant_) and any algae (estimating d_algal_), on any location on the planet, and even enables comparisons within and among these three groups. As examples, while the ZoNI is restricted to *Z. noltii* [46] and the POMI to *P. oceanica* [44], the d_grass_ allows comparisons among all seagrasses. Furthermore, it is possible to compare among seagrasses, algae and plants, estimating their distances to their respective IBLs, namely the d_grass_, d_algal_ and d_plant_;
iii. *Its background* The d_grass_ is grounded on the biomass–density relations of plants and algae, thoroughly studied since the 1950s with the self-thinning law accolade of “the only generalization worthy of the name law in plant ecology” [2]. It has already performed well when applied to other subjects. Its previous application to worldwide algal data has determined that the efficiency of space occupation in the algae depends on taxonomic group, functional group, clonality and latitude [12]. Its current application to seagrass worldwide has unveiled how it can be used to compare stands and interpret efficiency in light of intrinsic (population dynamics) and extrinsic (eco-physiological) factors.

## Conclusions

The placement of seagrass meadows in the biomass–density plot is limited by their interspecific boundary line (IBL) setting a maximum efficiency of space occupation. Furthermore, species tend to differentiate the bands each occupies in this scatter-plot, which is evidence of their conditional differentiation. The efficiency of space occupation by seagrasses, requiring only the biomass and shoot-density of their stands and measured by their perpendicular distance to the seagrass IBL, revealed a highly useful indicator of their ecological condition. It identified the summer as their most favourable season and the lower intertidal as their preferred depth. It discriminated among locations. It identified which nutrients were in excess, thus acting as pollutants, and beyond which concentrations did these have a deleterious effect over seagrasses. The d_grass_ was revealed to be a most efficient ecological index with general application and comparable with similar indexes developed for seaweeds and terrestrial plants.

## Additional files

**Additional file 1: Fig. S1.** Additional figure.

**Additional file 2.** The biomass and density presented in 32 studies on 10 seagrass species scattered worldwide. Additional biotic and abiotic variables are also provided depending on their availability from the respective study.

**Additional file 3.** The LOLS software fitting dgrass to the nutrients data.

## Abbreviations

B: biomass
D: density
d_algae_: perpendicular distance to the algae IBL
d_grass_: perpendicular distance to the seagrass IBL
D_plant_: perpendicular distance to the plant IBL
IBDR: interspecific biomass–density relationship
IBL: interspecific boundary line
PAR: photosynthetic active radiation
PCA: principal components analysis
PC1: principal component #1
POMI: *Posidonia oceanica* multivariate index
UBDL: ultimate biomass–density Line
w: individual plant weight
ZoNI: *Zostera noltii* multi-metric index
