# Supplementary figures and images for: The biomass–density relationship in seagrasses and its use as an ecological indicator
Source: BMC Ecol. 2018 Oct 19;18:44. doi: 10.1186/s12898-018-0200-1 (PMC6195692; doi:10.1186/s12898-018-0200-1)

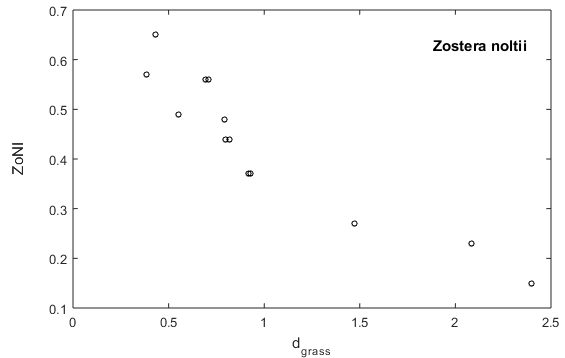

Supplement: Supplementary file 1 — Additional file 1: Fig. S1. Additional figure. [file 12898_2018_200_MOESM1_ESM.tif]
