# Supplementary material for: The biomass–density relationship in seagrasses and its use as an ecological indicator
Source: BMC Ecol. 2018 Oct 19;18:44. doi: 10.1186/s12898-018-0200-1 (PMC6195692; doi:10.1186/s12898-018-0200-1)
Supplement: Supplementary file 3 — Additional file 3. The LOLS software fitting dgrass to the nutrients data. [file 12898_2018_200_MOESM3_ESM.zip › Tutorial.docx]

TUTORIAL

The data and software run in Matlab ®. The **LOLS_single_fit.m** file runs the calculus using the data in the **SeagrassData.mat** file with the possible predictors (independent variables) and the dgrass response (dependent variable). The user may apply this software to other data sets. The **LOLS_single_fit.m** file has an initial **Settings** section were the user edits the analysis. The posterior Calculus setting is not to be edited by the user, unless he masters the respective numerical analysis and Matlab programming, and intends to change the way the calculus is implemented. Even though, changing the calculus is very risky and is advisable to first save an original version of the file.

In the **Settings** section, the Matlab file with the data and the directory were it is located are declared in line 7. If the variables were already loaded into Matlab workspace, the user should comment line 7 with the % symbol in its beginning. This way, Matlab bypasses this line considering it a no-code line. The predictor is declared in line 10 and the response in line 11. In this case, the predictor is the log10 of the ammonium concentration and the response is -dgrass so that its hump points upward. There were a few observations were dgrass was undetermined. Numerical undetermined values, represented in Matlab as NaN (Not-a-Number), contaminate and spread through the calculus leading everything beyong them to be also undetermined. Such observations must be eliminated from the input arrays, which was done by selecting only the ones that were not undetermined i.e., (~isnan(dgrass)).

The LOLS, the IRLS and the MLE require optimization methods to find the solutions. Here, is used the classical Newton-Raphson method. However, convergence to the true solution requires close enough initial guesses, which may be iteratively tried. The initial guesses are defined in lines 14, 17, 20 and 23. The search stoping criteria are defined in lines 33 and 36. Further settings specific of each method are declared in lines 39, 42 and 45.

The optimal parameter values are provided in the data structure ‘Results’.

The ‘OptEvol’ array gives the rate of convergence of the parameters estimation from time t to t+1. The time series is along each column, one column for each parameter.
